# Recent advances in the reconstruction of cranio-maxillofacial defects using computer-aided design/computer-aided manufacturing

**DOI:** 10.1186/s40902-018-0141-9

**Published:** 2018-02-05

**Authors:** Ji-hyeon Oh

**Affiliations:** Department of Oral and MaxilloFacial Surgery, Dental Hospital, Gangneung-Wonju National University, Gangneung, South Korea

**Keywords:** Computer-aided design/computer-aided manufacturing, Three-dimensional imaging, Cranio-maxillofacial defect, Reconstructive surgical procedures, Custom implant, Patient-specific implant

## Abstract

With the development of computer-aided design/computer-aided manufacturing (CAD/CAM) technology, it has been possible to reconstruct the cranio-maxillofacial defect with more accurate preoperative planning, precise patient-specific implants (PSIs), and shorter operation times. The manufacturing processes include subtractive manufacturing and additive manufacturing and should be selected in consideration of the material type, available technology, post-processing, accuracy, lead time, properties, and surface quality. Materials such as titanium, polyethylene, polyetheretherketone (PEEK), hydroxyapatite (HA), poly-DL-lactic acid (PDLLA), polylactide-co-glycolide acid (PLGA), and calcium phosphate are used. Design methods for the reconstruction of cranio-maxillofacial defects include the use of a pre-operative model printed with pre-operative data, printing a cutting guide or template after virtual surgery, a model after virtual surgery printed with reconstructed data using a mirror image, and manufacturing PSIs by directly obtaining PSI data after reconstruction using a mirror image. By selecting the appropriate design method, manufacturing process, and implant material according to the case, it is possible to obtain a more accurate surgical procedure, reduced operation time, the prevention of various complications that can occur using the traditional method, and predictive results compared to the traditional method.

## Introduction

The reconstruction of complex cranio-maxillofacial defects is challenging due to the unique anatomy, the presence of a vital structure, and the variety of deficits [1, 2]. The reconstruction of congenital or acquired cranio-maxillofacial defects due to congenital abnormalities, post-trauma, tumor resection, and infection requires both functional and esthetic considerations [3, 4].

Computer-aided design (CAD) is the process of creating, modifying, analyzing, or optimizing a design using computer system. Computer-aided manufacturing (CAM) is the process of planning, managing, or controlling manufacturing using computer system [5].

**Fig. 1:**
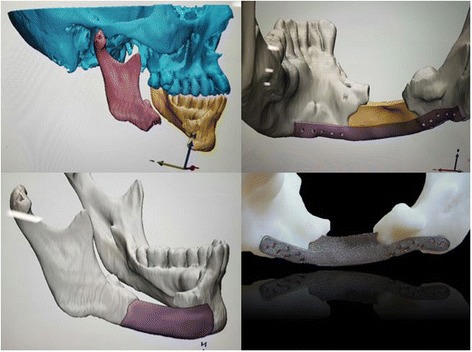
Pre-operative diagnosis, virtual surgery, and creation of patient-specific implants using CAD/CAM software

In the early 1960s, CAD was developed in the aircraft and automotive industries, and in the late 1960s, the supply of computer system for CAD was specialized [6]. In the late 1980s, computer-controlled milling was used to produce prosthesis using three-dimensional (3D) imaging data in computed tomography (CT) [7]. With the development of CAD/CAM technology, there have been increasing cases of restructuring cranio-maxillofacial defects to improve appearance and function with more accurate surgery and shorter operation times [8]. With CAD/CAM software, accurate pre-operative planning can be established, and surgeons can perform virtual ablation, plan osteotomy and reconstruction procedures, or create patient-specific implants (PSIs) [3, 4, 9] (Fig. 1).

The advantages of CAD/CAM technology include improved accuracy of esthetic results, restoration of large and geometrically complex anatomical defects, reduction of operative times, more accurate fitting of implants, overcoming the disadvantages of autogenous bone grafts, and performing resection and reconstruction in one step [10, 11]. The 3D printing technique in the cranio-maxillofacial area surgery includes contour models that are accurate replicas of patient-specific anatomy, guides that are patient-specific templates that guide precise cutting and drilling, splints defined as the replica of the virtual post-operative position of the patient structure, and implants defined as three-dimensionally printed objects that are directly implanted in the patients [12, 13].

In this paper, we will discuss the manufacturing processes using CAD/CAM, implant materials, the workflow reconstructing the cranio-maxillofacial defects, and future directions of development.

## Review

### The manufacturing processes

The manufacturing processes include subtractive manufacturing, which cuts off a piece of material to form the final shape, and additive manufacturing, which builds up the material by stacking [8]. Subtractive manufacturing, the traditional machining technique has the disadvantage in that it is difficult to make complicated shapes by computer numerical control (CNC) milling and there is a lot of material waste [14].

**Fig. 2:**
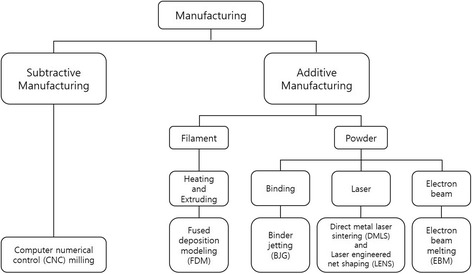
Schematic of the manufacturing processes

Additive manufacturing, known as rapid prototyping or 3D printing, has the advantage of being very sophisticated, with less material waste, faster production times, and the ability to produce complex structures [8]. There are several additive manufacturing processes, including binder jetting (BJG), direct metal laser sintering (DMLS), electron beam melting (EBM), laser engineered net shaping (LENS), and fused deposition modeling (FDM) [15–17]. Figure 2 is a simplified schematic of the manufacturing processes described in this paper.

**Fig. 3:**
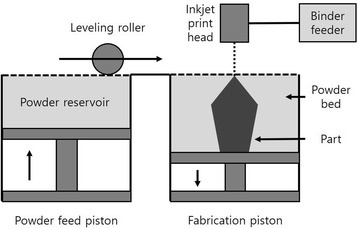
Binder jetting schematic

BJG generally uses two materials: a powder material from which the part is made and a binder material that bonds between the powder materials (Fig. 3). It has the advantage that parts can be produced without support structures, but it has the disadvantage that post-processing takes more time than actual printing, resulting in a significant increase in cost. In addition, the parts have rough microstructure and lower mechanical properties than those produced by selective laser melting (SLM) or EBM because of the possibility of porosity and heat treatment [16].

**Fig. 4:**
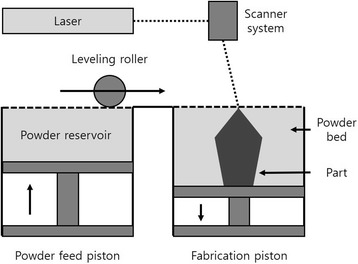
Direct metal laser sintering schematic

DMLS, referred to using the terms SLM or selective laser sintering (SLS), uses a high-powered optic laser to fuse the metal powder to solid components based on a 3D CAD file and, similar to EBM, is built layer by layer [17]. Similar to BJG, a powder bed is used to create a 3D object. However, instead of using a spray solution, a laser is used to tie the powder particles together, and the laser is instructed to draw a specific pattern on the surface of the powder bed during the printing process [18]. When the first layer is completed, the roller sprinkles a new layer of powder on top of the previous layer, pushes it flat, and then uses the laser to make the object layer by layer [19] (Fig. 4). DMLS have many advantages and disadvantages [16]. The advantages include the use of a wide range of materials, improved functionality, relatively low cost, and the production of ready-to-use near-net-shaped components. On the other hand, the disadvantages include relatively slow processes, size limitations, high power consumption, and high initial cost. In addition, the handling of the powder is tricky, the produced parts can have rough surfaces, and the brittle materials that cannot accommodate high internal stress during the manufacturing process can cause cracking of the parts.

**Fig. 5:**
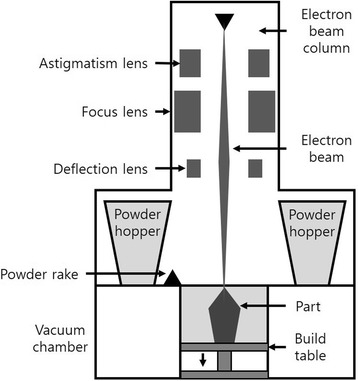
Electron beam melting schematic

EBM is very similar to DMLS, but there is a slight difference in that the parts are fabricated by melting the metal powder in a layer using an electron beam [17] (Fig. 5). In EBM, the cooling rate can be greatly reduced by increasing the temperature of the powder bed. Unlike DMLS, EBM has the ability to treat brittle materials that cannot be processed by DMLS because it slowly cools, avoiding solidification cracking of brittle materials. However, it takes longer than DMLS and requires sufficient cooling time before removing parts from the substrate plate because the electron beams are used many times in the layers [16].

**Fig. 6:**
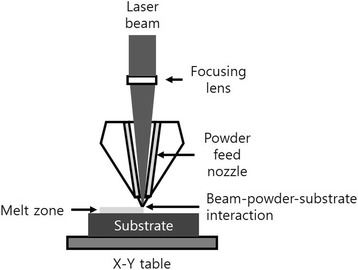
Laser engineered net shaping schematic

Similar to EBM and DMLS, LENS is used to fabricate metal parts directly from CAD solid models and has the difference in that metal powders are injected into the molten pool generated by the condensed high-power laser beam [17]. The molten material line rapidly solidifies as the laser beam retreats, and after each layer is formed, the laser head advances by one-layer thickness together with the powder feed nozzle, and a subsequent layer is created. This is repeated several times until the entire object displayed in the 3D CAD model is created [20] (Fig. 6).

**Fig. 7:**
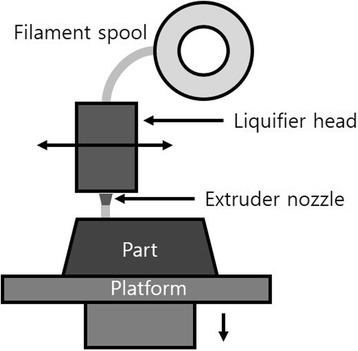
Fused deposition modeling schematic

FDM is generally carried out with a polymer melted in a printer nozzle and arranged layer by layer. The material is melted and deposited at a defined location on the printing layer, and after the first layer is completed, the distance between the printing bed and the extruder nozzle is increased and the second layer is printed on the first layer [21] (Fig. 7).

There are various kinds of manufacturing processes. The manufacturing process should be selected with consideration of the material type, available technology, post-processing, accuracy, lead time, properties, and surface quality [16].

### Implant materials

The ideal material is biocompatible, easy to shape, high strength, non-toxic, inexpensive, durable, radiolucent, and lightweight [8, 22]. However, no material satisfies these conditions [22–24]. Materials include non-resorbable materials such as titanium, polyethylene, polyether ether ketone (PEEK), and hydroxyapatite (HA) and absorbable materials such as poly-DL-lactic acid (PDLLA), polylactide-co-glycolide acid (PLGA), and calcium phosphate.

Titanium is the metal of choice for manufacturing implants. It has the advantages of high strength, biocompatibility, lightweight, corrosion resistance, and the potential for osseointegration [8, 25, 26]. However, it has the disadvantage of causing scatter artifacts in CT scans [27].

Polyethylene includes porous polyethylene (PPE) and ultra-high molecular weight polyethylene (UHMW-PE). PPE such as Medpor (Pufex Surgical Inc., College Park, GA, USA) was used for reconstruction of the orbital floor and augmentation of the facial area [28]. PPE is very stable and easy to shape and has tissue ingrowth through its pores [29, 30]. However, there is a possibility of infection [28]. UHMW-PE was used for reconstruction of orbit or temporomandibular joint by making PSIs using CAD/CAM [31, 32]. Because of a solid structure, UHMW-PE can have a lower infection rate than PPE [32]. Polyethylene has the advantage of not producing artifact because of radiolucency in CT, but it also has a disadvantage that it is difficult to control implant position after surgery [3, 32].

PEEK was used to reconstruct various craniofacial bone defects including cranioplasty [23, 33]. PEEK has similar strength and elasticity to bone and is easy to modify [34]. It is radiolucent in CT and offers more comfort to patients, with lower thermal conductivity and lighter weight than titanium. However, it had reports of infection and foreign body reaction [27].

HA is used as a biocompatible scaffolding material for bone tissue engineering [35]. It is osteoconductive and non-resorbable and shows tissue in-growth in the presence of pores, with a strong capacity to bind both hard and soft tissues [36]. Pure HA is low in viscosity and difficult to make complex shapes, but it can be overcome with custom-made HA using CAD/CAM [37, 38].

As absorbable implants, PDLLA and PLGA are commonly used, especially in pediatric craniofacial surgery [39]. However, foreign body reaction and the weakness of materials such as screw fracture have been reported [40, 41]. There are cases in which calcium phosphate implants have been used for the reconstruction of cranio-maxillofacial defects [42]. These printed calcium phosphate implants have good biocompatibility and suitable biodegradation and are similar to the mineral phase of the bone, so they do not cause artifacts or interference seen in other metallic alloplasts in CT or MRI. In addition, calcium phosphate implants show less mechanical performance than titanium but are suitable as a scaffold for bone tissue growth and can be loaded with bioactive protein or antibiotics.

### Workflow

The modeling software used for 3D printing includes Mimics (Materialise, Leuven, Belgium), SolidWorks (Dassault Systemes, Velizy-Villacoublay, France), Amira (FEI Visualization Sciences Group, Merignac, France), Rhino (Robert McNeel & Associates, Seattle, WA, USA), and SurgiCase CMF (Materialise, Leuven, Belgium). The printing software include ZPrinter and Projet (3D Systems, Rock Hill, SC, USA) and Alaris (Objet Limited, Rehovot, Israel) [12].

PSIs can be constructed through a manufacturing process and can also be produced by shaping directly from a 3D printing skull model [3]. In general, CT scan images are converted into two-dimensional (2D) digital imaging and communications in medicine (DICOM) files and converted to the 3D stereolithography (STL) format using CAD software [1, 8, 43]. The design methods for the reconstruction of the cranio-maxillofacial defects are as follows.

i) After printing the skull model of the pre-operative form, pre-bending of the plate, fabrication of an onlay template by model surgery, or fabrication of the implant on the skull model [44–48]
ii) After performing virtual surgery, including resection and reconstruction in the pre-operative image, printing of a resection guide or fabrication of the template by printing the skull model with virtual surgery [49, 50]
iii) After printing of the skull model reconstructed symmetrically using a mirror image of the unaffected side, pre-bending of the plate, using it as a template, or fabrication of the implant directly on the skull model [51–53]
iv) After reconstruction symmetrically using a mirror image of the unaffected side and design of the 3D implant to fit precisely to the defect, fabrication of the PSI by transferring the PSI data to CAM software [3, 8, 32, 54–57]

### Future directions

Digital workflows are time consuming and cannot be used for emergency procedures such as immediate post-traumatic surgery [10]. It takes from a few days to weeks to make the PSI outside the hospital [8]. However, with the development of 3D printers, relatively inexpensive personalized 3D printers have been introduced and the accuracy has increased, making it possible to manufacture products inside the hospital, reducing the time required. In addition, the development of professional CAD software familiar to surgeons and minimally invasive surgical procedures will provide predictable results.

## Conclusion

By selecting the appropriate design method, manufacturing process, and implant material according to the case, it is possible to obtain a more accurate procedure, reduced surgical time, the prevention of various complications that can occur using the traditional method, and predictive results compared to the traditional method.
